# Characteristics of MSCs in Synovial Fluid and Mode of Action of Intra-Articular Injections of Synovial MSCs in Knee Osteoarthritis

**DOI:** 10.3390/ijms22062838

**Published:** 2021-03-11

**Authors:** Ichiro Sekiya, Hisako Katano, Nobutake Ozeki

**Affiliations:** Center for Stem Cells and Regenerative Medicine, Tokyo Medical and Dental University, 1-5-45 Yushima, Bunkyo-ku, Tokyo 113-8510, Japan; katano.arm@tmd.ac.jp (H.K.); Ozeki.arm@tmd.ac.jp (N.O.)

**Keywords:** MSC, synovial fluid, mode of action, injection, synovium, knee, osteoarthritis, TSG-6, PRG-4, BMP-2

## Abstract

We have been studying mesenchymal stem cells (MSCs) in synovial fluid and the intra-articular injection of synovial MSCs in osteoarthritis (OA) knees. Here, mainly based on our own findings, we overview the characteristics of endogenous MSCs in the synovial fluid of OA knees and their mode of action when injected exogenously into OA knees. Many MSCs similar to synovial MSCs were detected in the synovial fluid of human OA knees, and their number correlated with the radiological OA grade. Our suspended synovium culture model demonstrated the release of MSCs from the synovium through a medium into a non-contacting culture dish. In OA knees, endogenous MSCs possibly mobilize in a similar manner from the synovium through the synovial fluid and act protectively. However, the number of mobilized MSCs is limited; therefore, OA progresses in its natural course. Synovial MSC injections inhibited the progression of cartilage degeneration in a rat OA model. Injected synovial MSCs migrated into the synovium, maintained their MSC properties, and increased the gene expressions of TSG-6, PRG-4, and BMP-2. Exogenous synovial MSCs can promote anti-inflammation, lubrication, and cartilage matrix synthesis in OA knees. Based on our findings, we have initiated a human clinical study of synovial MSC injections in OA knees.

## 1. Introduction

Mesenchymal stem cells (MSCs) are derived from mesenchymal tissue and have the functional capacity to self-renew and generate a number of differentiated progeny [1]. These cells participate in tissue homoeostasis, remodeling, and repair by ensuring the replacement of mature cells that are lost during the course of physiological turnover, senescence, injury, or disease [2]. MSCs can be isolated from bone marrow as well as from various adult mesenchymal tissues, including the synovium [3,4]. Reports on the intra-articular injection of MSCs for the treatment of knee osteoarthritis (OA), the most prevalent degenerative joint disease, have increased in recent years. The incidence of OA is rising due to the aging of populations [5], and OA currently has no effective disease-modifying drugs due to its complicated chronic pathology. However, a systematic review has shown that MSC injections can improve OA pain in many cases and increase cartilage volume in some cases [6,7,8]. We have been actively studying MSCs obtained from synovial fluid, as well as the intra-articular injection of these MSCs in OA knees. This paper provides an overview, based largely on our own findings, of the role of endogenous MSCs in the synovial fluid of OA knees and their mode of action when injected exogenously into the synovium as a therapy for OA knees.

## 2. Main

### 2.1. MSCs in Synovial Fluid of OA Knees

**Figure 1:**
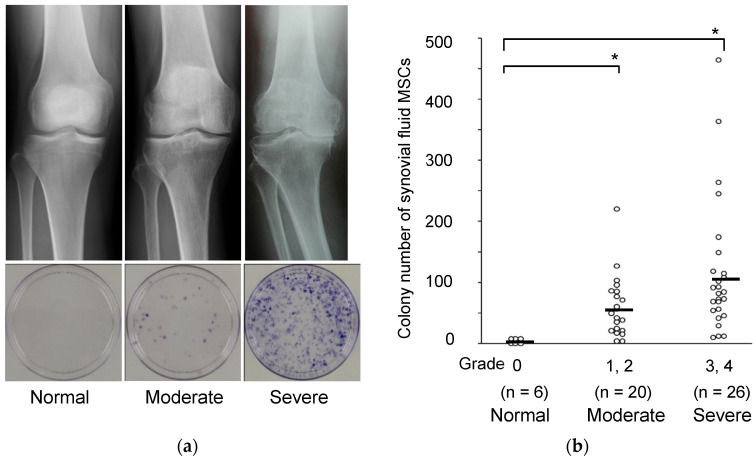
MSCs in synovial fluid derived from osteoarthritis patients by radiographic grade. (**a**) Radiographic images of the knees and representative dishes showing colonies of synovial fluid MSCs. (**b**) Relationship between the Kellgren–Lawrence grading and the colony number of synovial fluid MSCs per synovial fluid volume (mL). Average values are shown as bars (* *p* < 0.05) (reproduced from [9]).

We aspirated synovial fluid from OA knees, plated the cell components on a 10 cm-diameter dish, and cultured the cells for 14 days. Staining with crystal violet revealed a large number of cell colonies (Figure 1a). These colony-forming cells could differentiate into chondrocytes and adipocytes and undergo calcification, indicating a multi-differentiation potential [9]. Thus, the colony-forming cells from synovial fluid of OA knees had the characteristics of MSCs, and the number of colonies reflected the number of MSCs in the synovial fluid. MSCs in synovial fluid were seldom detected in normal volunteer knees but were found in much greater numbers in OA knees, and their number correlated with the radiological OA grade (Figure 1b) [9].

### 2.2. MSCs from Synovial Fluid Resemble Synovial MSCs

**Figure 2:**
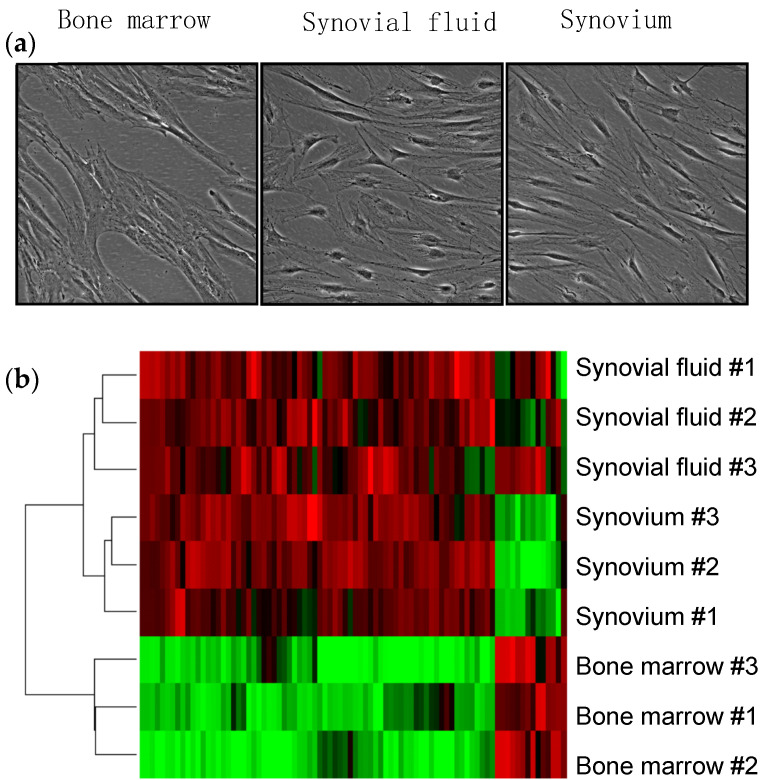
Comparison of synovium MSCs, synovial fluid MSCs, and bone marrow MSCs from osteoarthritis patients. (**a**) Representative morphologies. (**b**) Comparison of the gene expression profiles by hierarchical clustering analysis (reproduced from [9]).

We collected the synovial fluid, bone marrow, and synovium from the same OA knee during knee arthroplasty and prepared MSCs from those tissues. Morphologically, the synovial fluid MSCs appeared to be closer in character to synovial MSCs than to bone marrow MSCs, as the synovial MSCs and synovial fluid MSCs were narrower than bone marrow MSCs and their nuclei were more obvious (Figure 2a). We also prepared mRNA derived from each MSC type from three knees and conducted a comprehensive gene expression analysis using microarrays. Hierarchical clustering analysis demonstrated that the gene profiles in the synovial fluid MSCs were more similar to those of synovial MSCs than those of bone marrow MSCs (Figure 2b) [9]. These results indicate that the MSCs in synovial fluid are similar to the MSCs of the synovium.

### 2.3. Migration of MSCs from the Synovium via the Synovial Fluid in a Tissue Culture System

**Figure 3:**
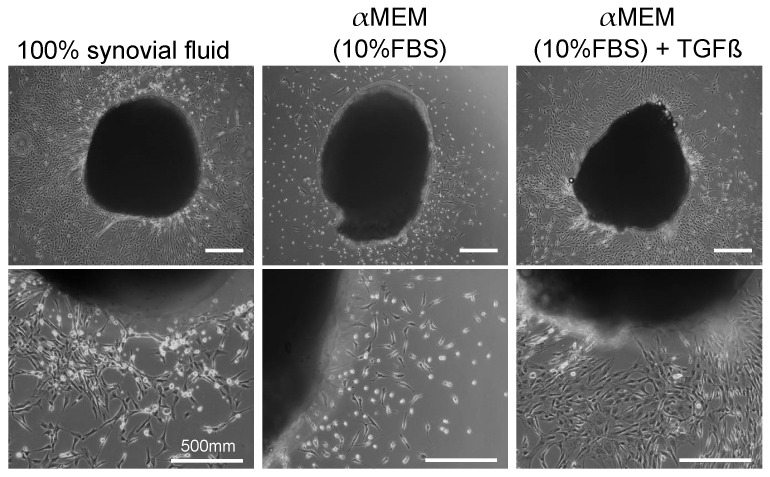
Morphology of an explant culture of synovium. Synovial tissues were cultured for 8 days in autologous synovial fluid, αMEM (10%FBS), or αMEM (10%FBS) with 10 ng/mL of TGFβ (reproduced from [10]).

Synovial fluid from OA knee contains MSCs. One of the possible reservoirs of the MSCs found in synovial fluid is the synovium itself, and synovial fluid may induce the mobilization of MSCs into synovial fluid in OA patients. We investigated whether synovial fluid could expand synovial MSCs in a tissue culture system. The synovial fluid in OA knees contains a high concentration of transforming growth factor β (TGFβ), so the effect of TGFβ was also examined. Autologous synovial fluid promoted a greater expansion of synovial MSCs than was observed with treatment with α minimum essential medium (αMEM) + fetal bovine serum (FBS), and the addition of TGFβ to αMEM+FBS increased this expansion to a similar level in samples from all 11 OA donors (Figure 3). The synovial cells expanded in synovial fluid retained their multipotentiality and showed surface markers characteristic of MSCs. The addition of an anti-TGFβ neutralizing antibody to the synovial fluid partially inhibited synovial cell expansion. These experiments demonstrated that autologous synovial fluid enhanced the expansion of MSCs in tissue cultures of synovium from OA patients by promoting cell migration, and this effect was partially induced by TGFβ [10].

### 2.4. MSCs Released from Suspended Synovium

**Figure 4:**
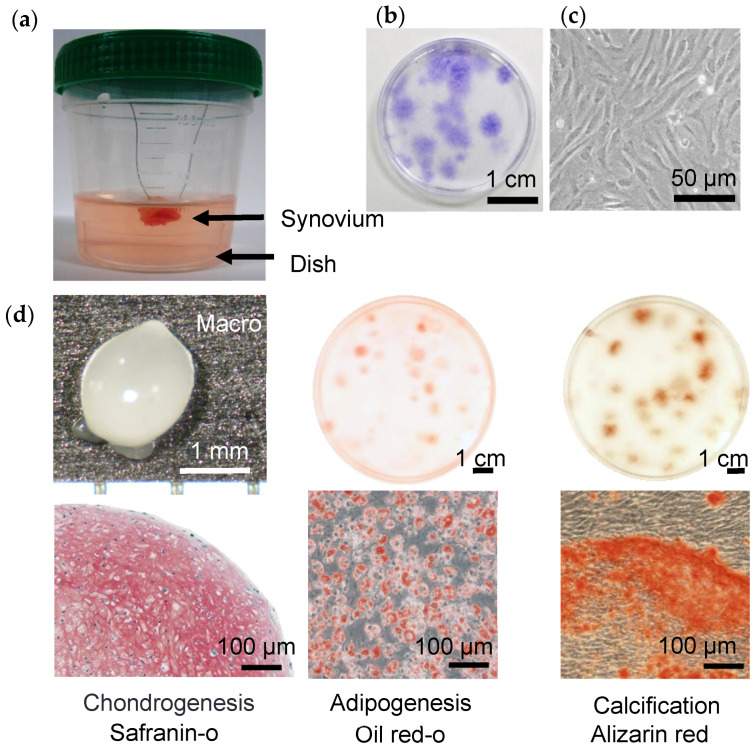
MSCs released from suspended synovium. (**a**) Suspended synovium culture model. Approximately 1 g of synovium was suspended in a bottle containing a culture dish at the bottom. (**b**) Cell morphology. After culturing for 7 days, the dish was observed. (**c**) Culture dishes stained with crystal violet. (**d**) Chondrogenesis, adipogenesis, and calcification (reproduced from [11]).

Although the origin of the MSCs found in synovial fluid could be the synovium, no direct evidence had been demonstrated to confirm that MSCs were mobilized from the synovium into the synovial fluid. We developed a novel in vitro model, the “suspended synovium culture model”, to provide this evidence. The synovium was harvested during total knee arthroplasty, cut into approximately 1 g specimens, and washed thoroughly to remove blood. Each synovium specimen was then sutured with 4-0 nylon thread and suspended in a 100 mL bottle. A 35 mm-diameter culture dish containing 40 mL αMEM+FBS had previously been placed at the bottom of the bottle. The specimens were cultured for 7 days (Figure 4a) and then stained with crystal violet staining. Cell colonies were confirmed from the specimens from all 28 donors (Figure 4b). The colony-forming cells consisted of spindle cells (Figure 4c), and they formed a cartilage pellet that was positively stained with safranin-o, differentiated into adipocytes, and calcified when cultured in the appropriate differentiation medium (Figure 4d). This suspended synovium culture model confirmed the release of MSCs and their passage through the medium in a bottle into a non-contacting culture dish [11].

### 2.5. MSCs in Synovial Fluid in Intra-Articular Tissue Injuries

We previously demonstrated that the MSCs in synovial fluid increased in number after anterior cruciate ligament (ACL) injury in humans [12]. Cluster analysis of gene profiles demonstrated that the synovial fluid MSCs from patients with ACL injury were more similar to synovial MSCs than to bone marrow MSCs, as previously observed in patients with OA [12]. We also attempted injection of synovial MSCs into the knee joint in a rabbit model of partial ACL. A greater number of injected MSCs was observed in the injured area than in the uninjured area of the ligament [12]. Kanaya et al. injected MSCs into the knees of rats with a partially resected ACL and demonstrated adhesion of the cells to the injured site and a contribution of MSCs to the repair of the resection [13]. 

We also examined the correlation between the number of MSCs in synovial fluid and the cartilage degeneration score evaluated arthroscopically in 22 patients with ACL injury. The MSC number in the synovial fluid was correlated with the cartilage degeneration score [9]. Use of a rabbit cartilage defect model also confirmed adhesion of injected synovial MSCs to the cartilage lesion and the promotion of cartilage regeneration [14].

MSCs in synovial fluid were also greater in number in knees with a meniscus injury than in normal knees. The number of MSCs in synovial fluid was positively correlated with the duration after meniscus injury [15]. The synovial MSCs injected into the knee joint adhered to the meniscus lesion and promoted meniscal regeneration in partial meniscectomy models in rats [16,17], rabbits [18], and pigs [19].

These findings suggest that synovium serves as a reservoir of MSCs that are mobilized following intra-articular tissue injuries and that migrate to the injury site to participate in the repair response. However, the number of endogenous MSCs in the synovial fluid is probably too small to contribute to the repair of intra-articular tissue injuries by natural processes.

### 2.6. Possible Roles of Endogenous MSCs in the Synovial Fluid of OA Knee

**Figure 5:**
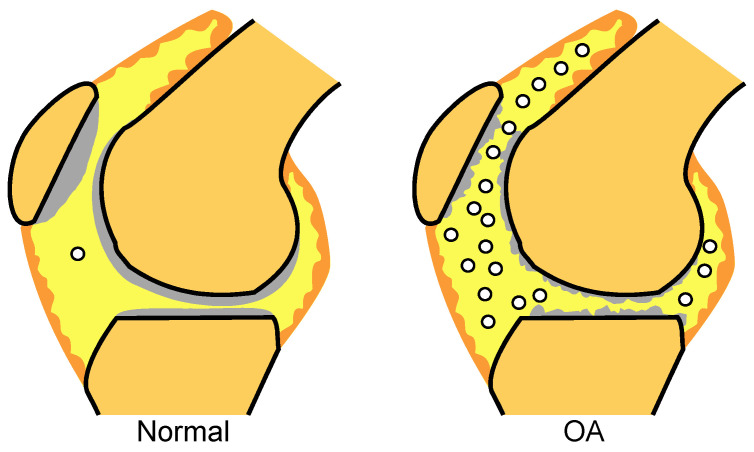
MSCs in synovial fluid. MSCs in synovial fluid are seldom detected in normal knees but are found in greater numbers in OA knees.

In the adult cartilage, enzymatic activities and the mechanical stress imposed on the joints inevitably lead to cartilage damage. Under normal circumstances, this damage is overcome by the turnover of matrix components synthesized by chondrocytes. Thus, in the normal adult articular cartilage, a balance exists between cartilage anabolism and catabolism. In OA, the catabolism becomes stronger than the anabolic capacities of chondrocytes; therefore, the cartilage matrix degenerates and the joint cartilage is damaged [20]. MSCs are rare in the synovial fluid in normal knees but are much more prevalent in OA knees (Figure 5). We speculate that the endogenous MSCs mobilize from synovium through synovial fluid and act protectively. However, the number of MSCs that can be mobilized is limited, so OA undergoes its natural progression. We therefore investigated whether the intra-articular injection of exogenous synovial MSCs could inhibit the progression of osteoarthritis.

### 2.7. Synovial MSC Injections in a Rat OA Model

**Figure 6:**
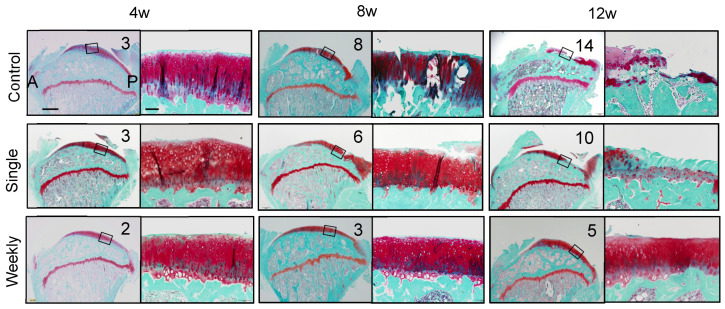
The effect of intra-articular injections of synovial MSCs in ACL-transected rats. Synovial MSCs were injected once or weekly. Histological sections of tibial cartilage stained with safranin-o and the Osteoarthritis Research Society International (OARSI) scores are shown. A, anterior; P, posterior (reproduced from [21]).

We completely transected the ACL of the rats and allowed them to walk freely in the cage. The rats were given intra-articular injections of PBS alone (control group) or 1 × 10^6^ synovial MSCs once (single group) or weekly (weekly group), beginning one week after the surgery. In the control group, the cartilage fissured at 8 weeks and extensively disappeared by 12 weeks (Figure 6). In the single group, the cartilage disappeared over time but was better preserved than in the control group. In the weekly group, the cartilage was essentially preserved at 12 weeks [21]. The MSC injections inhibited the progression of cartilage degeneration in a frequency-dependent manner.

### 2.8. Distribution of Synovial MSCs after Injection

**Figure 7:**
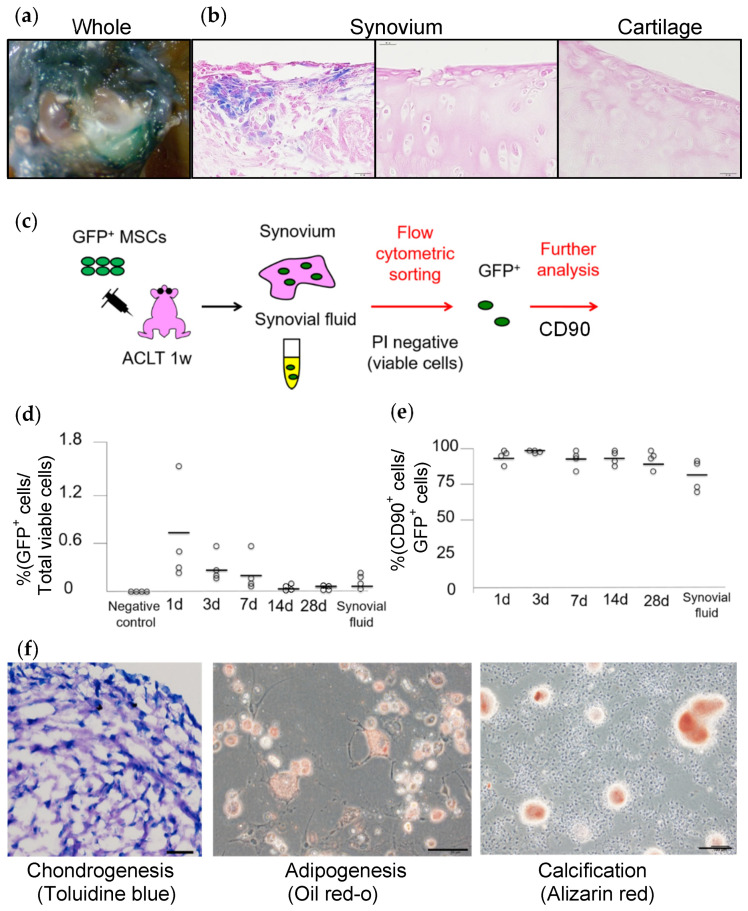
Distribution and properties of synovial MSCs injected into the knee. (**a**) The whole knee joint one day after injection of synovial MSCs expressing the LacZ gene into a rat knee. The ACL had been transected 7 days before. (**b**) Histological sections stained with X-gal. (**c**) Schema for the flow-cytometry assay. GFP-expressing MSCs were injected into the knees of rats 7 days after ACL transection and the synovium was harvested 1 week later. After enzymatic digestion, GFP+ cells were sorted for further analysis. PI, propidium iodide. (**d**) Ratio of GFP+ cells relative to total viable cells. (**e**) Ratio of CD90+ cells relative to total GFP+ cells. (**f**) Multi-differentiation potential of the sorted GFP+ cells 28 days after injection (reproduced from [21]).

We injected rat synovial MSCs expressing LacZ into the knee to determine the distribution of the cells at 1 day. The MSCs with blue staining for X-gal were widely observed in the synovium (Figure 7a) but not in the cartilage or meniscus (Figure 7b). We also injected luciferase-expressing rat synovial MSCs into the knee and analyzed them with an in vivo imaging system to investigate the activity of the cells and their migration out of the joint. The fluorescence was more prominent in the ACL-transected knee injected once than in the intact knee injected once, but the fluorescence was no longer detectable in either knee after 14 days. By contrast, a sustained fluorescence was observed after 14 days in the group that received weekly injections. The injected synovial MSCs were engrafted into the synovium, and weekly injections maintained a high cellular activity. The injected MSCs did not migrate outside the knee joint [21].

### 2.9. Surface Epitopes and Differentiation Potential of MSCs after Migration

We injected green fluorescent protein (GFP)-expressing MSCs (GFP+ MSCs) into the knee, selected GFP+ cells migrating into the synovium using flow cytometry, and examined the MSC properties of the migrated cells (Figure 7c). The percentage of GFP+ cells relative to the total viable cells decreased gradually over time (Figure 7d), but the percentage of CD90+ cells relative to the total GFP+ cells remained at approximately 90% after 28 days (Figure 7e). The GFP+ cells that were sorted after migrating into the synovium retained their multi-differentiation ability for chondrogenesis, adipogenesis, and calcification (Figure 7f) [21]. Therefore, the synovial MSCs injected into the knee joint and migrated to the synovium maintained the properties of MSCs.

### 2.10. Gene Expression of Synovial MSCs after Migration to Synovium

**Figure 8:**
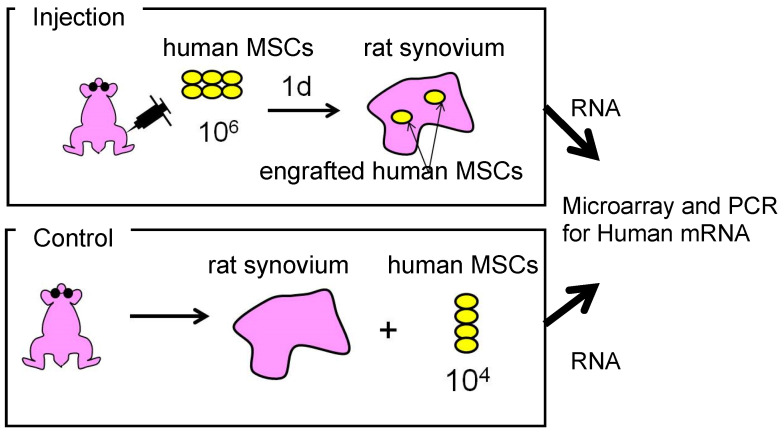
Scheme for species-specific gene expression analysis. We injected 1 × 10^6^ human synovial MSCs into the knees of rats 7 days after ACL transection. One day later, we harvested rat synovium and prepared total RNA for microarrays and RT-PCR. We also prepared total RNA from a mixture of harvested, un-injected control rat synovium, and 1 × 10^4^ human synovial MSCs as a control (reproduced from [21]).

We used species-specific gene expression to analyze the gene expression changes of human synovial MSCs that migrated to rat synovium. Approximately 1% of the 1 × 10^6^ human MSC injected into the rat synovium of knees with ACL transection had migrated one day after the intra-articular injection. The human transcriptomes were then compared between the MSCs in the synovium one day after injection and a control consisting of un-injected rat synovium mixed with 1 × 10^4^ human MSCs (Figure 8). An analysis of human mRNA using microarrays showed that 5 genes had undergone a more than 100-fold increase, 21 genes had a greater than 50-fold increase, and 255 genes had a greater than 10-fold increase in the human MSCs that had migrated to the rat synovium. Human proteoglycan-4 (PRG-4) and human bone morphogenetic protein-2 (BMP-2) were among the 10 most highly expressed human transcripts (Table 1). Further expression analysis of human mRNA by reverse transcription- polymerase chain reaction (RT-PCR) showed significant increases in the expression of human PRG-4, human BMP-2, human bone morphogenetic protein-6 (BMP-6), and human TNF-stimulated gene-6 (TSG-6) [21]. PRG-4, also known as lubricin, is produced by synovial cells or by superficial zone chondrocytes and has a key role in the homeostasis and maintenance of cartilage [22]. BMP-2 and BMP-6 have important biological effects on chondrocyte differentiation, cartilage matrix synthesis, and cartilage protection [23]. TSG-6 is secreted by transplanted MSCs and suppresses inflammation [24].

### 2.11. Mode of Action in Synovial MSC Injection Therapy for OA

**Figure 9:**
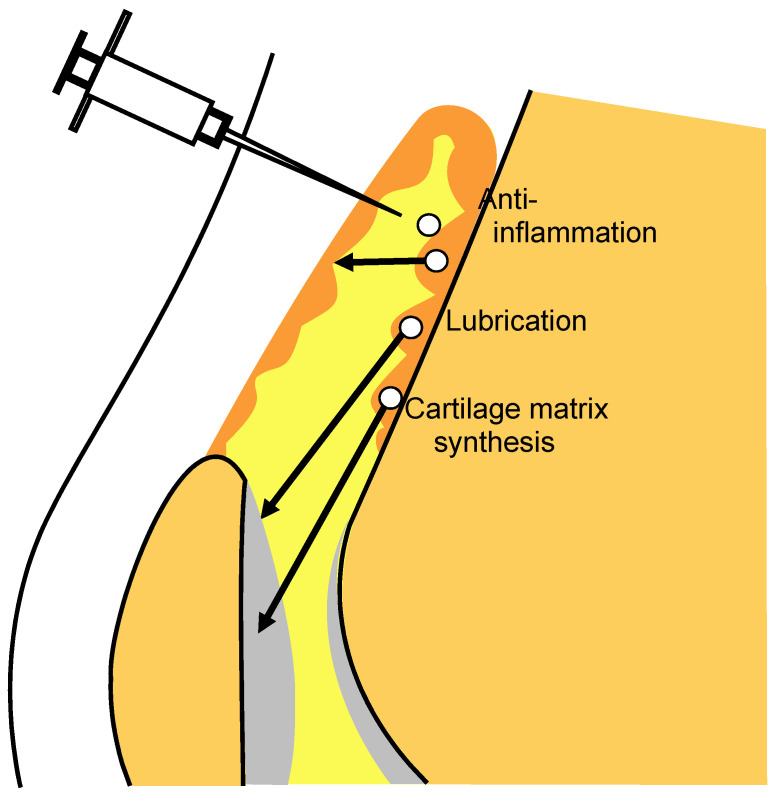
Mode of action in synovial MSC injection therapy for OA. Synovial MSCs injected into the knee joint migrate to the synovium and maintain the properties of MSCs. Synovial MSCs promote anti-inflammation, lubrication, and cartilage matrix synthesis. Consequently, synovial MSC injections inhibit the progression of OA.

Synovial MSCs injected into the knee joint mostly migrated to the synovium, and the cells maintained their MSC properties without differentiating into other lineages. Synovial MSCs act as anti-inflammatory agents through TSG-6 expression, as lubrication agents by PRG-4 expression, and in cartilage matrix synthesis by BMP expression (Figure 9). Consequently, exogenous MSCs can inhibit the progression of OA.

### 2.12. Clinical Study of Synovial MSC Injections into OA Knees

**Figure 10:**
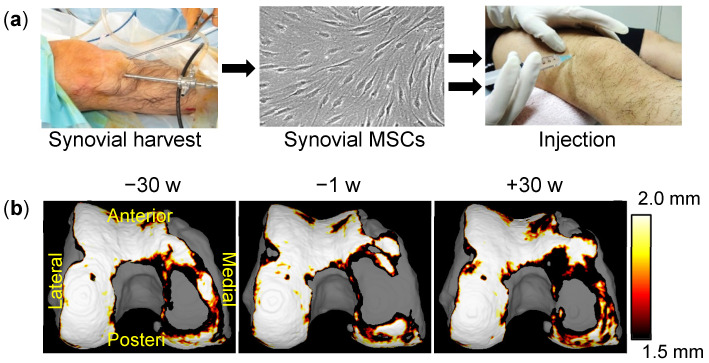
Clinical study of synovial MSC injections into OA knees. (**a**) Scheme of a clinical study of autologous synovial MSC injections into the OA knees of human patients. The synovium was harvested and enzymatically digested. The resulting synovial MSCs were expanded with autologous human serum and then injected into the knee twice at a 15-week interval. (**b**) Cartilage thickness mappings in a representative patient who underwent synovial MSC injections into a knee at time 0 and 15 weeks. MRI examinations were performed at −30, −1, and +30 weeks. Using the software we developed, the cartilage area was automatically extracted and visualized in three dimensions. Femoral cartilage at the posteromedial region at −30 weeks had decreased at −1 week but increased again at +30 weeks.

We have previously reported that synovial MSC transplantation into cartilage defects in human patients improved MRI findings and clinical scores [25], and that synovial MSC transplantation into a repaired meniscus improved clinical outcomes [26]. Based on the results of these clinical and preclinical studies, we started a novel clinical study, “Intra-articular injections of synovial stem cells for osteoarthritis of the knee (UMIN 000026732)”. to follow the effects of synovial MSC injections into osteoarthritic knees in 14 human patients. We harvested synovial tissue arthroscopically from each individual under local anesthesia, cultured the synovial MSCs with autologous serum, and injected two million cells into the knee twice at 15-week intervals (Figure 10a). In this clinical study, the last patient’s treatment has already been completed and we are currently in the process of analyzing the MRI and clinical outcomes. The preliminary results from fully automatic 3D MRI analysis indicate that some participants, who had shown decreased cartilage thickness in the posteromedial region of the femoral cartilage before injection, now show increased thickness after injection [27,28] (Figure 10b).

## 3. Concluding Remarks

In OA knees, MSCs may possibly mobilize from the synovium through synovial fluid and act protectively. However, the number of mobilized MSCs is limited, so OA progresses in its natural course. By contrast, synovial MSCs injected into the knee joint of a rat OA model migrate to the synovium, where they act as anti-inflammation, lubrication, and cartilage matrix synthesis agents, and inhibit the progression of OA. We have started a clinical study in which synovial MSCs are injected twice into the OA knees of human patients.

## Figures and Tables

**Table 1:** The top 10 human transcripts upregulated in the human MSCs that migrated within the synovium.

Gene Symbol	Gene Title	Fold Change
TFPI2	Tissue factor pathway inhibitor 2	252.6
PRG4	Proteoglycan 4	162.3
PTHLH	Parathyroid hormone-like hormone	130.5
T.L	Transcribed locus	107.5
LOC285359///PDCL3	Phosducin-like 3 pseudogene///phosducin-like 3	102.6
PLA2G4A	Phospholipase A2, group IVA (cytosolic, calcium-dependent)	92.5
BMP2	Bone morphogenetic protein 2	87.4
RPS4Y1	Ribosomal protein S4, Y-linked 1	75.5
CACNA1D	Calcium channel, voltage-dependent, L type, alpha 1D subunit	63.6
COL15A1	Collagen, type XV, alpha 1	63.3

## Data Availability

No new data were created or analyzed in this study.
